# Astrocyte States in Brain Aging and Neurodegeneration: At the Crossroads of Senescence and Reactivity

**DOI:** 10.1007/s11064-026-04709-7

**Published:** 2026-03-10

**Authors:** João Bastos Lima Pacca-Corrêa, Beatriz Martins Fernandes, Michele Siqueira, Raffaela Schafbenker, Gabriela Joras Baumart, Isabella Vivarini Damico, Flávia Carvalho Alcantara Gomes, Isadora Matias

**Affiliations:** Instituto de Ciências Biomédicas, Universidade Federal do Rio de Janeiro, Rio de Janeiro, RJ 21941-902 Brasil

**Keywords:** Astrocytes, Brain aging, Cellular senescence, Astrocyte reactivity, Neuroinflammation, Neurodegeneration

## Abstract

Brain aging involves progressive disruption of tissue homeostasis and susceptibility to neurodegenerative disorders. Within this context, astrocytes are key determinants of region-specific physiology, given their roles in metabolic support, synapse regulation, proteostasis, neuroinflammation, and blood-brain barrier maintenance. Aging is accompanied by broad transcriptional and functional remodeling in astrocytes, leading to the emergence of distinct cellular states that cannot be defined by classical morphological criteria alone. This review discusses how aging modifies astrocyte identities toward reactive and senescence-like states. We summarize core features of astrocyte senescence, including altered secretory signaling, impaired neuronal support, and changes in mitochondrial and proteostatic pathways, while integrating recent single-cell and regionally transcriptomic studies that delineate multiple reactive states associated with aging and pathological contexts. We further address evidence that reactivity and senescence are not mutually exclusive endpoints, but may coexist, arise sequentially, or partially overlap depending on timing, brain region, biological sex, and pathological insults. Finally, we define key open questions and experimental priorities required to establish the temporal and causal relationships among astrocyte states. We argue that resolving these issues is essential for advancing therapeutic strategies that specifically target defined astrocyte phenotypes, rather than nonspecifically suppressing astrocyte activity, in aging and neurodegenerative diseases.

## The Aging of the Central Nervous System

The nervous system is responsible for movement, sensation, emotional responses, communication, cognition, information processing, and memory [1]. The human brain contains approximately 86 billion neurons and a roughly equal number of non-neuronal (mostly glial) cells [2]. Glial cells are broadly classified into microglia and macroglia according to their embryonic origin. Microglia arise from the mesoderm, whereas macroglia are derived from the neuroepithelium and include astrocytes, radial glia, oligodendrocytes, oligodendrocyte progenitor cells (OPCs, also known as NG2-glia), ependymal cells, and, outside the CNS, Schwann cells [3, 4]. Astrocytes, the central focus of this review, demonstrate a remarkable functional diversity, acting not only as supportive cells for neurons but also as sculptors of their activity.

Aging is a multifaceted biological process characterized by an accumulation of damage over time that gradually undermines cellular and tissue homeostasis, leading to functional decline and increased vulnerability to disease [5]. In the CNS, aging is associated with a progressive decline in cognitive functions, including processing speed, memory, executive function, and learning capacity [6]. Concurrently, it increases susceptibility to a range of neuropathological conditions, most notably neurodegenerative diseases (NDD) such as Alzheimer’s disease (AD), Parkinson’s disease (PD), and Amyotrophic Lateral Sclerosis (ALS) [7, 8]. Currently, over 55.2 million people are living with dementia worldwide, a number projected to rise to 78 million by 2030, with most cases occurring in low- and middle-income countries [9, 10]. Given the global increase in life expectancy and the socioeconomic burden of age-related diseases, elucidating the cellular mechanisms underlying brain aging has become a central focus of contemporary research [11–13].

The changes observed in the aging CNS can be categorized into structural, functional, cellular, and molecular alterations that collectively contribute to progressive cognitive decline [14]. Structurally, the brain undergoes a reduction in volume and weight, ventricular enlargement, widening of sulci, and gray matter atrophy, primarily due to decreased dendritic arbor complexity and neuronal shrinkage rather than massive neuronal loss [15, 16]. White matter deterioration is also evident, resulting from myelin degradation, axonal degeneration, and oligodendrocyte loss [14]. Additionally, age-related changes in the interaction between the brain parenchyma and the cerebrovascular system have been reported [17], together with increased blood-brain barrier (BBB) permeability due to endothelial cell dysfunction [18–20].

At the cellular and circuit levels, the loss of dendritic spines and synapses in the mammalian brain is most prominently observed in the hippocampus and prefrontal cortex (PFC) [21–23]. These changes do not occur uniformly across the brain. Instead, the CNS displays regionally and temporally distinct patterns of vulnerability to both aging and NDD. Although the mechanisms underlying this selective vulnerability are only beginning to be elucidated [24], emerging evidence highlights glial cells as key determinants of these region-specific trajectories.

Transcriptomic studies in mouse models have demonstrated that aging is associated with widespread astrocytic transcriptional remodeling across multiple brain regions [25, 26]. These age-associated programs are characterized by downregulation of genes related to core astrocytic homeostatic functions, together with upregulation of immune and complement signaling pathways, indicating major shifts in astrocytic gene signatures and the acquisition of distinct phenotypic states [27]. Importantly, human studies corroborate these findings, showing that brain aging is accompanied by profound transcriptional changes in glial cells, particularly astrocytes and oligodendrocytes, within regions highly vulnerable to aging and neurodegeneration, such as the hippocampus and substantia nigra [28]. Moreover, single-nucleus transcriptomic analyses across the aging-Alzheimer’s disease continuum reveal marked region- and stage-specific shifts in astrocyte states that are detectable during aging and become progressively exacerbated with disease progression [29], raising the possibility that astrocytic transcriptional shifts may predict regional brain vulnerability to age-related diseases.

Importantly, aging is also accompanied by the progressive accumulation of senescent cells, including glial cell populations, which contribute to chronic inflammation, tissue dysfunction, and increased vulnerability to NDD [18]. In the aging brain, these processes occur alongside widespread transcriptional and functional changes in astrocytes, creating a permissive environment for the emergence of distinct astrocytic phenotypes. This raises fundamental questions regarding whether astrocytes preferentially adopt reactive phenotypes, senescent programs, or overlapping cellular identities, and how these states influence regional brain vulnerability and disease susceptibility during aging.

In this review, we provide a comprehensive overview of astrocyte heterogeneity across the lifespan, with particular emphasis on how aging modifies astrocytic physiology and identities. We discuss the molecular and functional features of astrocyte senescence and reactivity, examine points of convergence and divergence between these processes, and highlight emerging concepts and open questions relevant to brain aging and NDD.

## Astrocytes: Central Roles in CNS Homeostasis and Vulnerability during Aging

Astrocytes were originally described by Virchow (1858) as passive elements whose limited role was thought to be providing trophic and structural support to neurons. Although this view persisted for decades, it is now well established that astrocytes perform a wide array of essential and dynamic functions critical for maintaining neural circuit integrity in the healthy brain. These include: (1) regulation of extracellular ionic homeostasis; (2) control of metabolic substrate transport in response to neuronal activity via neurovascular coupling [30, 31]; (3) production of trophic soluble factors that help preserve BBB integrity [32, 33]; (4) transfer of functional mitochondria to neurons and endothelial cells to ensure cell survival and BBB integrity [34, 35]; (5) promotion of neuronal survival; (6) guidance of axonal growth; and (7) support of synapse formation, maturation, and refinement [36, 37].

Beyond these fundamental roles, astrocytes exhibit remarkable morphological, molecular, and functional heterogeneity. Classically, they are divided into protoplasmic astrocytes in gray matter, characterized by highly branched processes that envelop synapses, and fibrous astrocytes in white matter, which show longer, less ramified processes aligned with axonal tracts. Additional subtypes exist across specialized CNS regions, such as Bergmann glia in the cerebellum, Müller cells in the retina, and radial glia-like cells in neurogenic niches [38]. Human astrocytes, in particular, display increased size, complexity, and transcriptional diversity compared to rodent astrocytes, suggesting species-specific adaptations that may influence cognition and circuit modulation [39, 40].

Aging introduces additional layers of complexity to astrocyte biology. Several studies have demonstrated that astrocytes undergo structural, metabolic, and transcriptional changes across the lifespan, even in the absence of overt pathology. These alterations may emerge gradually and reflect adaptations to cumulative metabolic stress, evolving neuronal demands, and shifts in the extracellular environment. For instance, aging impacts astrocyte morphology in a region-dependent manner. In the substantia nigra, astrocytes in the pars compacta of aged mice show increased branching and volume, whereas astrocytes in the pars reticulata do not exhibit similar changes [41]. In contrast, hippocampal astrocytes in the CA1 region of aged mice display dystrophic features, including reduced process complexity and decreased cell volume, while CA3 astrocytes retain a more complex morphology [42]. These findings underscore that astrocytes do not follow a uniform aging trajectory; rather, their structural adaptations may depend on regional microenvironmental demands and vulnerabilities.

Transcriptomic studies in both mice and humans have consistently shown that astrocyte aging involves broad, coordinated transcriptional remodeling in a region-dependent and evolutionarily conserved manner [27]. Notably, while neuron-specific genes remain relatively stable across several brain regions during human aging, astrocyte- and oligodendrocyte-enriched transcriptional programs exhibit marked age-related shifts, particularly within the hippocampus and substantia nigra [28]. In mice, Boisvert et al. (2018) further demonstrated that astrocytes display distinct regional aging signatures, with hypothalamic and cerebellar astrocytes undergoing more extensive transcriptional changes than cortical astrocytes, especially in pathways related to synapse elimination, immune responses, and cholesterol metabolism [25]. Complementing these observations, Clarke et al. (2018) showed that aging induces pronounced, region-specific transcriptional remodeling in astrocytes, with hippocampal and striatal populations exhibiting far more extensive changes than their cortical counterparts. These shifts include a coordinated downregulation of genes involved in core homeostatic functions, such as metabolic support, mitochondrial maintenance, and antioxidant defense, together with the upregulation of genes typically associated with inflammatory or reactive responses [43], a topic that will be further discussed in subsequent sections.

Together, these findings support the view that aging modifies astrocyte identity and homeostatic capacity, thereby influencing how these cells respond to subsequent stressors or pathological insults. Importantly, however, the functional consequences of many of these age-associated transcriptional changes remain incompletely understood.

Addressing this gap, Lee et al. showed that aging is associated with the emergence of a specific astrocyte population, termed autophagy-dysregulated astrocytes (APDAs; discussed further below), which display impairments in key astrocyte-mediated processes, including reduced secretion of molecules that support synapse formation, as well as a diminished capacity to engulf and degrade synaptic material, consistent with compromised lysosomal function [44]. In line with these findings, our group has recently shown decreased levels of Hevin, an astrocyte-derived glycoprotein involved in synapse formation, in astrocytes from AD mouse model [45]. Together, these observations highlight how aging-associated astrocyte dysfunction may contribute to synaptic vulnerability and cognitive decline in NDD.

Extending these observations, we further demonstrated that astrocyte senescence is accompanied by a significant loss of synaptogenic and neuritogenic capacity in vitro, suggesting aging-related changes in neuron–astrocyte interactions and circuit-supporting functions provided by astrocytes [46]. This deficit is paralleled by profound mitochondrial alterations, as senescent astrocytes and astrocytes from aged brains exhibit increased mitochondrial fragmentation, reduced mitochondrial membrane potential, impaired biogenesis and altered fusion–fission dynamics, consistent with a state of astrocytic metabolic stress [47, 48]. Importantly, however, not all astrocytic homeostatic functions are uniformly downregulated during aging or senescence; in contrast to the reduced synaptic support, components of the glutamate-glutamine cycle are upregulated in senescent astrocytes, as well as in the hippocampus of aged mice and in human aged brain tissue, suggesting the activation of compensatory metabolic mechanisms aimed at preserving neurotransmitter homeostasis [49].

Collectively, these findings indicate that aging is associated with a complex set of morphological, molecular and functional changes in astrocytes, characterized by both loss and compensation of homeostatic functions. Such remodeling likely creates a permissive landscape for the emergence of distinct astrocytic states, including cellular senescence and reactive phenotypes, which will be explored in the following sections.

## Cellular Senescence: Concepts, Hallmarks and Relevance to Astrocyte Biology

### Cellular Senescence

Cellular senescence refers to a stable cellular state characterized by a permanent arrest of the proliferative cycle and by distinctive molecular, metabolic and phenotypic alterations that impact physiological processes across different organs and tissues [50, 51]. Additionally, it is recognized as one of the 12 hallmarks of aging [5], reflecting its broad contribution to age-associated functional changes. Beyond its classical definition in dividing cells, accumulating evidence indicates that post-mitotic populations, such as neurons, as well as cell types with low proliferative rates, including glial cells, can acquire senescence-like features that compromise cellular function and promote epigenetic and molecular alterations, although the underlying mechanisms and markers may differ among cell types [12, 52, 53].

Cellular senescence can be triggered by a wide range of intrinsic and extrinsic stressors, including telomere attrition, DNA damage, oxidative stress, proteostatic imbalance, oncogene activation, hypoxia, and mitochondrial dysfunction [54, 55]. These insults engage distinct signaling pathways, giving rise to replicative, oncogene-induced, and stress-induced senescence programs [56, 57]. As a consequence, senescent cells display heterogeneous morphological, metabolic, transcriptional, and secretory features that vary with cell type, tissue context, and the nature of the initiating stimulus, both in vitro and in vivo [58]. This heterogeneity is reflected in differences in the composition of the senescence-associated secretory phenotype (SASP), the kinetics of senescence induction, and chromatin remodeling, including the formation of senescence-associated heterochromatin foci [59, 60].

Despite this variability, senescent cells share recurring features, including persistent DNA damage signaling with accumulation of proteins such as 53BP1 and phosphorylated histone H2AX; increased lysosomal beta-galactosidase (β-Gal) activity; morphological and nuclear alterations linked to cytoskeletal modifications; reduction of lamin‑B1, and cell cycle arrest due to increased expression of p16^INK4a^ and p21^WAF1/Cip1^, which inhibit cyclin‑dependent kinases (CDKs) via repression of E2F target genes [51].

A central hallmark of senescence is the SASP, characterized by the secretion of pro-inflammatory cytokines such as IL-6, IL-8, TGF-β, and IL-1β, as well as growth factors and proteases [61]. Through paracrine and autocrine signaling, the SASP reinforces senescence locally and propagates senescence-associated programs to neighboring cells. At the molecular level, nuclear factor κB (NF-κB) acts as a key transcriptional regulator of SASP components, sustaining inflammatory gene expression and stabilizing the senescent state [62]. In parallel, increased production of reactive oxygen species (ROS) further amplifies NF-κB–dependent signaling, establishing self-reinforcing inflammatory loops that contribute to chronic tissue dysfunction [63].

Beyond its molecular and morphological hallmarks, senescence is a dynamic and context-dependent cellular program. It contributes to physiological processes such as embryonic development, wound healing and tissue remodeling, in part through immune recruitment and immunomodulatory signaling. However, when senescent cells accumulate and persist, they disrupt tissue homeostasis by sustaining chronic inflammation, impairing intercellular communication, and limiting regenerative capacity [57, 64]. Within the CNS, senescent glial cells, including astrocytes, have emerged as particularly relevant contributors to aging-associated dysfunction.

### Astrocyte Senescence

The accumulation of senescent cells increases with age and has been linked to cognitive decline and vulnerability to NDD [18]. Among glial populations, astrocytes are particularly relevant due to their abundance and central role in CNS homeostasis. Astrocyte senescence has been consistently associated with aging-related inflammatory states and functional impairment [12, 40].

Historically, the study of astrocyte senescence was constrained by the lack of appropriate experimental models, a challenge further exacerbated by the diversity and complexity of senescent phenotypes. More recently, a range of experimental strategies such as replicative stress, oxidative and proteostatic challenges, DNA damage and chemotherapeutic insults have been used to induce senescence-like states in murine and human astrocytes [46, 65, 66]. These approaches have provided important insights into how senescence-related pathways intersect with astrocyte dysfunction, neuroinflammation and aging-associated neuropathology [67].

**Fig. 1:**
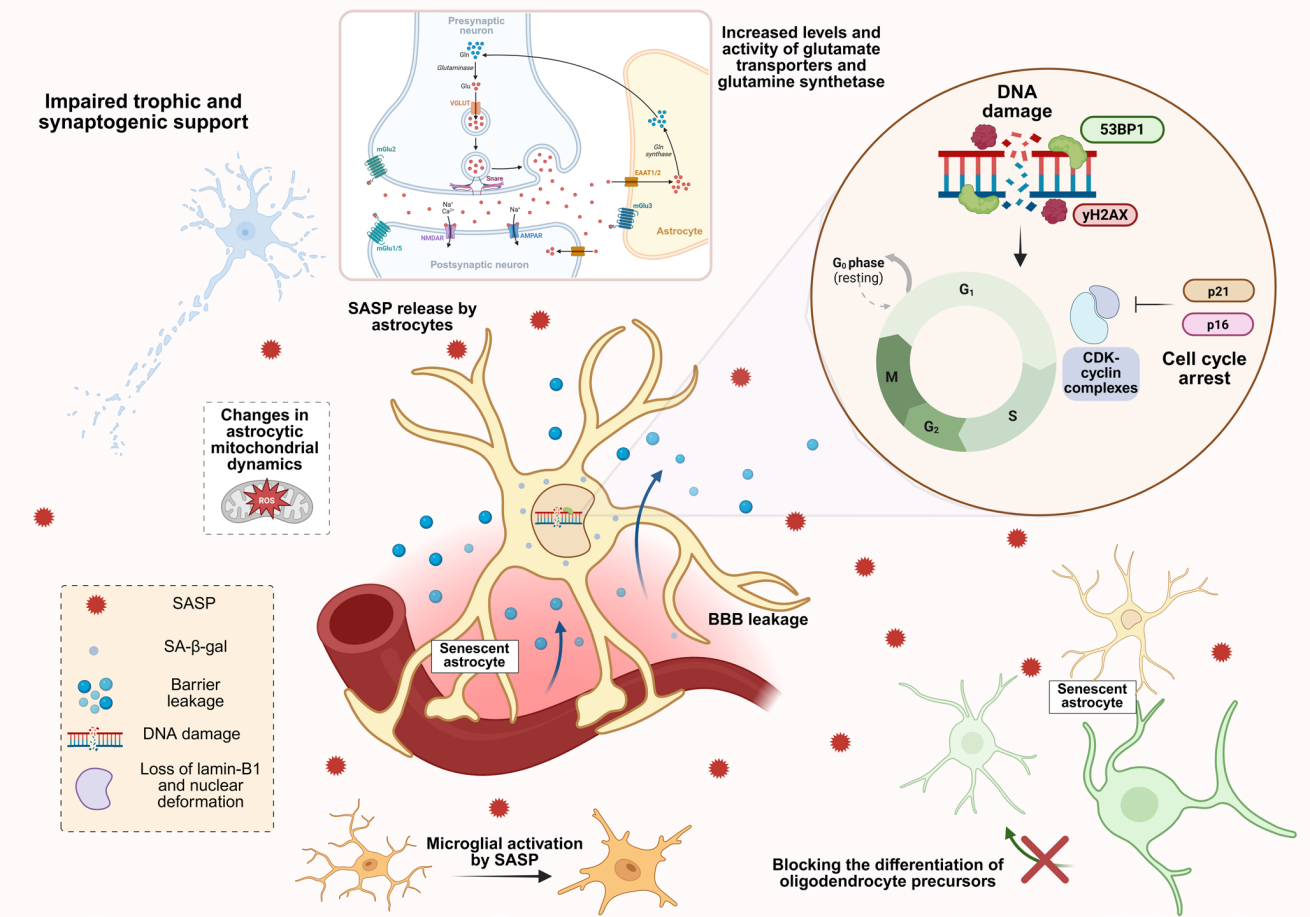
Astrocyte senescence: hallmarks and functional consequences. Schematic overview of key molecular and cellular features associated with astrocyte senescence, including persistent DNA damage signaling (53BP1 and γH2AX), cell-cycle arrest mediated by p16 and p21 inhibition of CDK-cyclin complexes, increased senescence-associated β-galactosidase activity (SA-β-gal), and SASP release. The figure also highlights major functional outputs linked to senescent astrocytes, such as impaired trophic/synaptic support, microglial activation, blood-brain barrier (BBB) leakage, altered mitochondrial dynamics, changes in glutamate metabolism (transporters and glutamine synthetase), and impaired oligodendrocyte precursor differentiation. Created with BioRender

Senescent astrocytes display classical senescence-associated features, including increased lysosomal content and β-gal activity, upregulation of cell-cycle arrest markers, such as p21^WAF1/Cip1^, p16^INK4a^ and p53, activation of persistent DNA damage responses, altered SASP profiles, and pronounced morphological changes [12]. In addition, our group has identified nuclear deformation and loss of lamin-B1 as robust and conserved hallmarks of astrocyte senescence in both murine and human models [46]. Importantly, in vivo evidence suggests senescence-associated features are not uniformly expressed across tissues, but instead emerge in a heterogeneous and context-dependent manner, strongly influenced by the local microenvironment and intercellular interactions [68]. A schematic overview of the molecular, cellular, and functional features associated with astrocyte senescence is presented in Fig. 1.

Functionally, the progressive accumulation of senescent astrocytes in aged neural tissue is predicted to compromise CNS homeostasis through altered secretion of inflammatory molecules and gene-regulatory factors [67]. Experimental evidence from our group indicates that senescent astrocytes exhibit a marked reduction in their capacity to promote neurite outgrowth and synapse formation, consistent with impaired neuritogenic and synaptogenic support to neurons [46]. Conversely, senescent astrocytes exhibit increased expression and activity of glutamate transporters and glutamine synthetase, suggesting compensatory mechanisms that may limit glutamate excitotoxicity in the aging brain, despite broader functional decline [49].

In parallel, astrocyte senescence has been associated with hypertrophy and increased GFAP expression [69], reflecting cytoskeletal changes that may favor pro-inflammatory factor production [70]. Complementary evidence from human pluripotent stem cell–derived astrocytes indicates that senescence is accompanied by cellular and nuclear morphological alterations, impaired mitochondrial function, reduced neurotransmitter clearance, and diminished capacity to support neuronal homeostasis [71]. Furthermore, Lye et al. (2019) identified a senescence-associated transcriptional signature in aged human astrocytes in vitro and reported that expression of a subset of these genes, including GFAPα, p14ARF, and TAU3, in peripheral blood correlated with mild cognitive impairment, suggesting that astrocyte senescence–related changes may also be detectable systemically and correlates with cognitive decline [72].

Given the extensive interactions between astrocytes and other CNS cells, senescence-associated alterations may compromise neighboring cells and fundamental cellular processes. Senescent astrocytes can negatively influence oligodendrocyte progenitor differentiation and myelination during aging [73]. Although astrocyte-derived extracellular vesicles can promote OPC differentiation under physiological conditions, vesicles from senescent astrocytes display altered protein content and fail to exert this supportive effect [73]. Moreover, it has been shown that factors secreted by senescent astrocytes can activate microglial cells and promote neuronal death via a pro-inflammatory secretory phenotype, as demonstrated in BV2 and N2a cell culture models [74]. BBB dysfunction has emerged as another relevant axis, although the bidirectional relationship between astrocyte senescence and BBB impairment remains incompletely defined. In this context, recent studies suggest that aging-associated BBB dysfunction may induce astrocyte senescence via TGF-β signaling triggered by albumin entry into the brain parenchyma [75].

At the mechanistic level, several evidence indicates that disruptions in mitochondrial quality control and proteostasis represent central hubs in the emergence and maintenance of the senescent astrocyte phenotype [76]. Senescent astrocytes exhibit increased mitochondrial fragmentation, impaired mitophagy, and heightened susceptibility to metabolic stress, despite upregulation of mitochondrial biogenesis as a potential compensatory response [47, 48]. In addition, silencing of clusterin, a chaperone involved in autophagic and endocytic pathways for misfolded protein clearance, induces senescence-like features in astrocytes, accompanied by increased ROS production and loss of proteostasis [77]. Together, these findings underscore the tight coupling between mitochondrial dysfunction, impaired proteostasis, and astrocyte senescence during aging.

Beyond aging, astrocytes expressing the senescence marker p16^INK4a^ have been reported in AD human brains and in mouse models of tau-dependent neurodegeneration [78, 79]. In PD, senescence-associated astrocytic phenotypes have been linked to dopaminergic neuron vulnerability, supported by increased p16^INK4a^ expression, decreased lamin-B1, mitochondrial dysfunction, and pro-inflammatory secretory signaling in midbrain astrocytes [80]. Similarly, in ALS, senescent astrocytes appear to compromise metabolic and trophic support to motor neurons while sustaining chronic inflammatory signaling [81]. Collectively, these findings suggest that astrocyte senescence represents a recurrent yet regionally patterned feature of brain aging and neurodegeneration, with important implications for selective neuronal vulnerability.

Considering the broad range of molecular, cellular, and tissue-level consequences associated with astrocyte senescence (Fig. 1), increasing attention has been directed toward additional cellular programs that influence astrocyte function and morphology during aging and NDD. Among these is astrocyte reactivity, described as a heterogeneous phenotype marked by changes in gene expression and morphology [82, 83]. Although conceptually distinct, senescence and reactivity share molecular, morphological and functional features, and growing evidence suggests they may coexist or influence one another depending on timing, brain region, and pathological context.

## Astrocyte Reactivity: Diversity of States in Aging and Disease

Astrocyte reactivity is a long-recognized cellular response to disturbances in CNS homeostasis and was initially defined based on morphological changes observed in injured or diseased brain tissue, including astrocytic hypertrophy, process remodeling and increased expression of intermediate filament proteins such as GFAP. Early neuropathological descriptions focused largely on acute insults, such as trauma, ischemia and infection; however, over time, the concept of astrocyte reactivity expanded to encompass coordinated transcriptional, molecular and functional changes [84].

In the context of aging and NDD, reactive astrocytes have emerged as a prominent and sustained feature, closely linked to chronic inflammation, synaptic dysfunction and progressive tissue vulnerability [40, 85]. Importantly, recent advances in transcriptomic and single-cell approaches have fundamentally reshaped this field, revealing that astrocyte reactivity does not represent a single uniform phenotype but rather a heterogeneous and dynamic continuum of states that vary across brain regions, disease stages and microenvironmental cues [82]. Over the past decade, and particularly in the last five years, omics-based studies have demonstrated that reactive astrocyte programs in aging and neurodegeneration extend well beyond classical classifications, highlighting astrocyte reactivity as a context-dependent process with diverse functional consequences [86].

Early investigations into astrocytic responses led to the classification of two major reactive phenotypes, termed A1 and A2, characterized by differences in morphology, gene expression profiles, and functional properties. A1 astrocytes were initially described as neurotoxic, as they lose the capacity to support neuronal viability and synaptic integrity, and contribute to BBB disruption [87]. In contrast, A2 astrocytes, typically induced in response to ischemia, exhibit neuroprotective features by secreting various trophic factors that promote neuronal survival and tissue repair [87].

During aging, astrocytes have been reported to acquire an A1-like reactive phenotype, as evidenced by the upregulation of genes with potential neurotoxic effects in different brain regions of aged mice [43]. These include components of the complement cascade (C3, C4B), the pro-inflammatory chemokine CXCL10, and the serine protease inhibitor Serpina3n. A1-like astrocytes have also been identified in several NDD, including AD, PD, Huntington’s disease, ALS, and multiple sclerosis [87–90]. These observations raise the possibility that astrocytic responses to aging may contribute to the increased vulnerability of the aged brain to neurodegeneration. Interestingly, the same study also reported increased expression of several A2-associated genes in the aged brain [43], suggesting that astrocytic states during aging extend beyond the oversimplified A1/A2 dichotomy and may involve mixed or transitional phenotypes.

Within this expanded conceptual framework, recent studies have begun to identify specific reactive astrocyte states that arise in response to distinct cellular stressors, particularly those linked to intracellular homeostatic imbalance. In this context, autophagy-dysregulated astrocytes (APDAs) have been described as a reactive astrocyte state characterized by morphofunctional alterations, including accumulation of autophagosomes inside swollen processes, impaired proteostasis, disrupted autophagic–lysosomal flux, and reduced synaptogenic capacity, yet notably lacking the upregulation of inflammatory and reactive genes typically observed in A1-like astrocytes [44]. The emergence of APDAs in aging and early in APP/PS1 mice suggests that astrocyte reactivity can be driven by cell-intrinsic stress pathways, especially those related to protein quality control, rather than by overt inflammatory cues [44]. In line with this notion, growing evidence indicates that alterations in astrocytic proteostasis are a central feature of NDD and contribute to synaptic vulnerability and circuit dysfunction, supporting the idea that dysregulated proteostasis represents a relevant axis of astrocyte reactivity in aging and age-related diseases [45].

Interestingly, the emergence of a distinct reactive astrocyte states has also been demonstrated in age-related diseases. By using single-nucleus RNA sequencing (snRNA-seq), an astrocyte subpopulation termed disease-associated astrocytes (DAAs) was identified in AD mouse models, aged WT mice, and the aged human brain [91]. DAAs are a unique subtype of GFAP-high reactive astrocytes, displaying a gene set associated with endocytosis, the complement cascade, and aging [91]. Among upregulated genes, DAAs express high levels of cathepsin B (*Ctsb*), a lysosomal protease involved in amyloid precursor protein (APP) degradation [92], as well as increased levels of *Serpina3n*, which has been associated with impaired amyloid clearance and increased Aβ accumulation [91, 93], and *Apoe*. The *Apoe* gene, under pathological conditions, loses its neuroprotective function and impairs Aβ protein clearance, contributing to toxic plaque deposition [94]. In this context, this astrocyte subpopulation was found located adjacent to Aβ plaques in AD and appears prior to manifestation of cognitive decline, increasing with disease progression [91]. This gene expression profile suggests a deleterious astrocytic role in amyloid accumulation. Although Ctsb and Apoe might initially act in response to neurotoxic aggregates, they fail to promote effective clearance, and elevated Serpina3n further exacerbates Aβ deposition.

More recently, a study described a distinct astrocyte subtype known as lipid-accumulated reactive astrocytes (LARA) in patients with temporal lobe epilepsy (TLE) and in murine models of the disease [95]. These astrocytes exhibit increased Apoe expression, associated with enhanced lipid droplet accumulation. Furthermore, LARA astrocytes show upregulation of genes involved in lipid transfer and metabolism, such as CLU, PTGDS, and PLCG2, and an enrichment of lipid transport-related pathways, presenting a distinct reactive astrocyte profile [95]. Similar to the mechanism proposed in AD, Apoe accumulation in this astrocyte subtype contributes to a neurotoxic profile by exceeding its lipid-processing capacity, leading to neuronal loss and disease progression.

Importantly, Sadick et al. further described nine different astrocytic populations in the brain of AD patients, each exhibiting unique transcriptomic phenotypes that extend beyond a simple neuroprotective or neurotoxic classification. Instead, these subtypes play crucial roles, such as synaptic support, as evidenced in subpopulations 0, 4, and 8, which express genes involved in synaptic assembly, organization, and transmission [96]. Together, these findings highlight that astrocyte responses in AD encompass a spectrum of specialized states with distinct roles in circuit maintenance, tissue remodeling, and disease progression, rather than converging toward a single reactive phenotype.

Furthermore, evidence indicates that inflammatory signaling represents a major axis driving astrocyte reactivity across aging and neurodegenerative contexts. A well-characterized mechanism involves cytokines released predominantly by activated microglia, including interleukin-1α (IL-1α), tumor necrosis factor (TNF) and the complement component C1q, which together are sufficient to induce robust inflammatory reactive programs in astrocytes [87]. At the intracellular level, these cues converge on NF-κB signaling, triggering broad transcriptional reprogramming and reshaping astrocytic immune, metabolic and synapse-related pathways.

Recent work by Leng et al. provided a more refined view of inflammatory astrocyte reactivity by identifying two interacting inflammatory states in human iPSC-derived astrocytes exposed to IL-1α, TNF and C1q. These states, termed inflammatory reactive astrocyte signatures 1 and 2 (IRAS1 and IRAS2), are distinguished by their downstream signaling pathways rather than by the classical A1/A2 framework. IRAS1 is predominantly associated with IL-1/IL-6-STAT3 signaling and exhibits features of an acute inflammatory response, characterized by increased expression of genes such as C3. By contrast, IRAS2 arises in the context of TNF-driven interferon signaling mediated by STAT1/2 and IRF1, leading to the induction of interferon-stimulated genes, including IFIT3, as well as inflammatory mediators such as VCAM1 and CXCL10, which are more commonly linked to sustained or chronic inflammatory responses [97]. Notably, these two inflammatory programs are not fixed or mutually exclusive. Instead, they exert reciprocal regulatory effects: activation of IL-6-STAT3 signaling favors IRAS1 while constraining interferon-dependent IRAS2 responses, whereas interferon signaling biases astrocytes toward the IRAS2 state while attenuating IRAS1 features. This bidirectional regulation illustrates the plastic nature of inflammatory astrocyte reactivity and indicates that astrocytes can shift between inflammatory states in response to changes in the strength, duration and composition of upstream signals [97].

In addition to cytokine-mediated pathways, inflammatory astrocyte reactivity is influenced by systemic signals and non-cell-autonomous mechanisms. In models of systemic inflammation induced by lipopolysaccharide (LPS), adenosine has emerged as a critical mediator capable of crossing the BBB and activating adenosine receptors on astrocytes. This activation triggers intracellular pathways including JAK-STAT3, MAPK and Fos-Jun signaling, thereby promoting astrocyte reactivity and reinforcing neuroinflammatory cascades through interactions with microglia [98]. Taken together, these observations indicate that inflammatory astrocyte reactivity reflects the convergence of local immune cues and systemic homeostatic perturbations rather than a single, isolated signaling route.

**Fig. 2:**
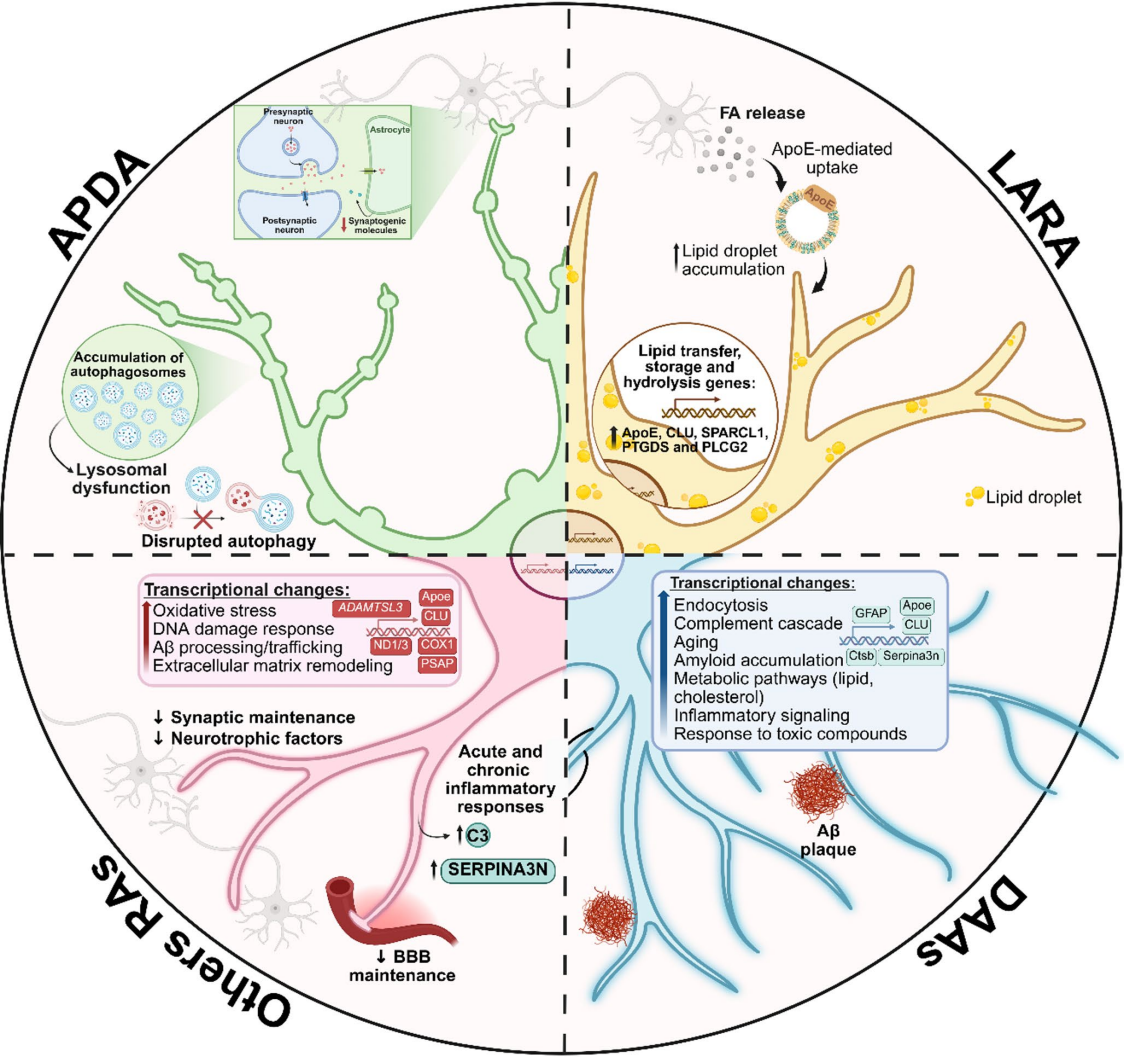
Reactive astrocyte states in aging and disease. Conceptual representation of distinct reactive astrocyte states reported in aging- and disease-related contexts. Autophagy-dysregulated astrocytes (APDA) are characterized by disrupted autophagic–lysosomal flux and reduced synaptogenic support. Lipid-accumulated reactive astrocytes (LARA) exhibit ApoE-associated lipid droplet accumulation and upregulation of lipid transfer/storage pathways. Disease-associated astrocytes (DAAs) show transcriptional enrichment in endocytosis, complement and inflammatory signaling, and are depicted in association with amyloid pathology. “Other RAs” (reactive astrocytes) summarize reactive profiles linked to oxidative stress, DNA damage responses, extracellular matrix remodeling, reduced BBB maintenance, and acute or chronic inflammatory signatures. Created with BioRender

Collectively, these findings underscore that astrocyte reactivity cannot be reduced to a single molecular signature or functional outcome. Instead, reactive states arise as dynamic and context-dependent responses that reflect the nature of the insult, intracellular stress pathways and local microenvironmental cues (Fig. 2). Importantly, reactive astrocyte phenotypes may coexist with, precede, or partially overlap other aging-associated astrocyte states, including cellular senescence. Distinguishing between these intersecting phenotypes remains a central challenge for the field and is critical for understanding how astrocytes contribute to brain aging and NDD progression.

## Reactivity vs. Senescence: Overlap, Implications and Future Directions

Astrocytes are highly heterogeneous cells whose identities reflect developmental origin, regional specialization and local circuit demands. This diversity is preserved throughout life and becomes increasingly apparent during aging and neurodegenerative conditions, as astrocytes respond to cumulative metabolic stress, altered neuronal activity and changes in the extracellular milieu. Recent work has reinforced the view that astrocyte phenotypes are better described as dynamic cellular states, defined by coordinated molecular and functional programs that vary according to context, rather than as fixed cell types [84, 99].

**Fig. 3:**
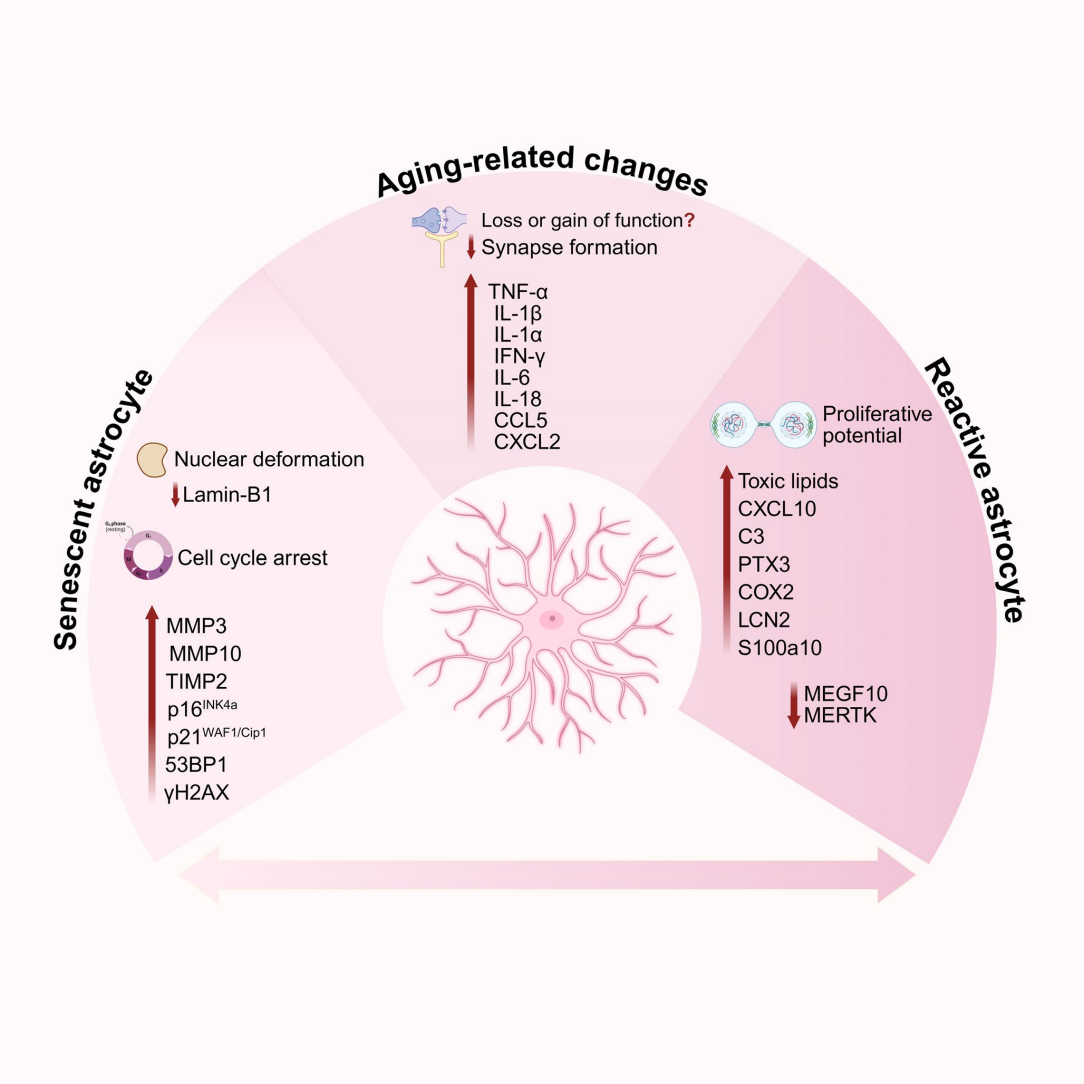
Reactivity and senescence as intersecting astrocyte states. Integrative model contrasting canonical features of senescent and reactive astrocytes and their shared, aging-related outputs. The schematic highlights senescence-associated hallmarks (nuclear deformation, lamin-B1 loss, cell-cycle arrest, and DNA damage markers) alongside reactive signatures (inflammatory mediators, complement-related genes, lipid-associated toxicity, and altered phagocytic/synapse-related factors). The top sector summarizes aging-related inflammatory cues and functional changes (including reduced synapse formation), emphasizing partial overlap and context-dependent convergence between reactivity and senescence. Created with BioRender

In this view, both astrocyte reactivity and senescence represent components of a broader spectrum of astrocyte states, encompassing different degrees of inflammatory signaling, metabolic adjustments, proteostatic imbalance and alteration of homeostatic functions across aging trajectories and disease contexts (Fig. 3). Notably, although astrocyte states are becoming increasingly well defined, additional layers of complexity are only beginning to be appreciated. Recent work highlights that astrocyte responses to aging and pathology are strongly influenced by biological sex and brain region, with distinct transcriptional and functional programs emerging in a context-dependent manner [100].

Importantly, reactive and senescent astrocytes should not be regarded as mutually exclusive identities. Instead, they may coexist within the same tissue, arise sequentially, or partially overlap depending on local conditions and disease stage. This perspective helps reconcile the heterogeneity observed across experimental models and human studies, suggesting that susceptibility to NDD depends not only on the presence of astrocyte dysfunction, but also on how specific astrocyte states interact with neuronal circuits, other glial cells, immune responses and systemic factors. Moving forward, approaches that combine spatially resolved transcriptomics, longitudinal analyses and functional analyses of defined astrocyte populations will be essential to clarify the temporal and regional dynamics of these states.

From a translational standpoint, we argue that recognition of the diversity and plasticity of astrocyte states highlights the need for therapeutic strategies aimed at modulating specific astrocytic pathways or programs, rather than broadly suppressing astrocyte reactivity or senescence in the aging and diseased brain.

## Data Availability

No datasets were generated or analysed during the current study.
